# Assessing the suitability of formalin-fixed paraffin-embedded (FFPE) tissue for genome-wide association studies (GWAS)

**DOI:** 10.1186/s13104-025-07306-z

**Published:** 2025-07-01

**Authors:** Julian Reingruber, Maximilian J. Krämer, Jessica Bigge, Pouria Dasmeh, Sandeep Grover, Stefanie Heilmann-Heimbach, Markus M. Nöthen, Nicole Kreuser, Ines Gockel, Lothar Veits, Michael Vieth, Paul Jank, Carsten Denkert, Carlo Maj, Timo Hess, Johannes Schumacher

**Affiliations:** 1 Center for Human Genetics, Philipps University Marburg and University Hospital Marburg, Marburg, Germany; 2 Mildred Scheel Early Career Centre (MSNZ) for Cancer Research Würzburg, University Hospital Würzburg, Würzburg, Germany; 3 Institute of Human Genetics, University and University Hospital Bonn, Bonn, Germany; 4 Next Generation Sequencing Core Facility, Medical Faculty, University Bonn, Bonn, Germany; 5 Department of Visceral, Transplant, Thoracic and Vascular Surgery, University Hospital of Leipzig, Leipzig, Germany; 6 Institute of Pathology, Friedrich-Alexander-Universität Erlangen-Nürnberg, Klinikum Bayreuth, Bayreuth, Germany; 7 Institute of Pathology, Philipps University Marburg and University Hospital Marburg, Marburg, Germany

**Keywords:** FFPE tissue, DNA extraction, Genome-wide genotyping, GWAS

## Abstract

**Objective:**

The power of genome-wide association studies (GWAS) to identify common disease variants depends primarily on the number of included samples. The availability of formalin-fixed paraffin-embedded (FFPE) samples in pathology institutes provides a valuable resource for GWAS, but the use of this material poses significant challenges. To explore the suitability of utilizing FFPE tissue for GWAS, we analysed the genotyping concordance between corresponding FFPE and blood samples. We evaluated both microarray technology and low-coverage whole-genome sequencing (lcWGS) to determine whether there were differences between genotyping methods.

**Results:**

In our concordance study, FFPE tissue showed high recall and precision values across both genotyping methods when compared to matched blood samples for single nucleotide polymorphisms. This demonstrates that FFPE samples are suitable for GWAS and that both methods are viable options for genotyping. However, microarray technology outperformed lcWGS, as evidenced by significantly higher recall (*p* = 0.005) and precision (*p* = 0.003) values. This, together with the lower cost of genotyping and computational efficiency, makes microarray technology currently the superior method for GWAS using FFPE tissue. Nevertheless, lcWGS has shown reliable results and holds the potential to provide more comprehensive and unbiased genetic variant analysis across diverse populations in the future. Our results show that the large number of FFPE samples stored in pathology institutes can significantly increase the power of future GWAS.

**Supplementary Information:**

The online version contains supplementary material available at 10.1186/s13104-025-07306-z.

## Introduction

In recent years, genome-wide association studies (GWAS) have provided important insights into the genetic architecture of multifactorial diseases [1]. By analysing common genetic variants across the genome in large case-control cohorts, GWAS facilitate the identification of disease-associated risk variants. The success of detecting these variants is highly dependent on the sample size included in the study [2]. Recruiting such samples, especially in the required numbers, can be difficult and time-consuming. This is particularly true for retrospective recruitment designs for life-threatening diseases, as many patients will have died shortly after diagnosis.

Formalin-fixed paraffin-embedded (FFPE) tissues have been stored in pathology institutes for decades and represent a powerful pool of readily available GWAS samples. While FFPE tissue is easy to use for microscopic analysis, working with DNA extracted from these tissues is challenging. Formalin fixation can cause DNA fragmentation and base modifications, such as deamination of cytosine to uracil. This can lead to false variant detection and reduce the yield of amplifiable DNA templates [3]. Despite these challenges, a growing number of research groups are attempting to implement genetic methods using FFPE DNA, indicating its high scientific value [4–6].

The aim of this study was to evaluate the use of FFPE tissues for GWAS by comparing genotype concordance between FFPE-derived DNA and corresponding blood-derived DNA. We employed both microarray technology and low-coverage whole genome sequencing (lcWGS) for genotyping. While microarrays are the standard for GWAS, the decreasing cost of sequencing combined with higher resolution and improved detection of rare genetic variants has generated significant interest in the use of lcWGS as an alternative [7].

The successful application of FFPE-derived DNA in GWAS could significantly expand the availability of samples for genetic association studies. Ultimately, this could lead to a better understanding of disease mechanisms.

## Materials and methods

### Sample collection

Formalin-fixed, paraffin-embedded, tumour-free oesophageal mucosa biopsies were obtained from the Institute of Pathology, University of Erlangen-Nürnberg, Bayreuth, Germany. Blood samples from corresponding probands were obtained from the Department of Visceral, Transplant, Thoracic and Vascular Surgery at the University Hospital of Leipzig, Germany. A total of three patients diagnosed with oesophageal adenocarcinoma (EA) were included in the study, and both FFPE and blood samples were collected from each patient. FFPE tissues were formalin-fixed in 2021 according to a standardized protocol using 4% neutral buffered formalin for 24 h. All FFPE samples were stained with haematoxylin-eosin (HE) to confirm the absence of tumorous tissue microscopically. The FFPE blocks were stored at room temperature in a dark and dry environment.

Genomic DNA from three Genome in a Bottle (GIAB) samples was obtained from the Coriell Institute for Medical Research (Camden, New Jersey, USA). To account for patient heterogeneity, samples from three distinct genetic backgrounds (East Asian, Ashkenazi Jewish, and European) were selected. The reference variant calling files were sourced from the GIAB consortium [8].

### DNA isolation

DNA isolation from blood samples was carried out using the Smart Blood DNA Midi Direct Prep Kit by AnalytikJena (Jena, Germany) on the InnuPure^®^ C16 touch system by AnalytikJena (Jena, Germany). For DNA isolation from FFPE samples, the biopsies were visually inspected to identify the tissue within the paraffin blocks. A disposable scalpel was used to extract approximately 5–10 mg of tumour-free tissue from each paraffin block, which was then transferred into reaction tubes. DNA isolation was performed using the Maxwell^®^ FFPE Plus DNA Kit by Promega (Madison, Wisconsin, USA). After DNA extraction, samples were subjected to quality control (QC) using the NanoDrop One (Thermo Fisher Scientific, Waltham, Massachusetts, USA) to assess DNA purity, the QuantiFluor^®^ ONE dsDNA System with Quantus™ Fluorometer (Promega, Madison, Wisconsin, USA) to quantify DNA concentration, and the TapeStation 4200 with gDNA Kit (Agilent Technologies, Santa Clara, California, USA) to evaluate DNA integrity. After completion of the QC procedures, the DNA samples were stored at a temperature of -20 °C until they were thawed for genotyping analyses.

### Microarray

Microarray genotyping was performed at Life&Brain GmbH (Bonn, Germany) using the Infinium Global Screening Array-24 v. 3.0 chip (Illumina, San Diego, California, USA). Genotyping was conducted according to Illumina’s standard protocols. Clustering and data pre-processing were carried out at the Institute of Human Genetics at the University of Bonn (Bonn, Germany). The data were subsequently processed into PLINK format files for further analysis [9].

QC procedures included the application of rigorous filtering criteria. Variants were retained if they met the following pre-imputation thresholds: missing call rate < 0.03, X-chromosomal F-statistic > 0.2 for females and < 0.8 for males, standardized F-statistic < 6 × standard deviation, KING-coefficient < 0.0884, Missing genotype rate < 0.03, and Hardy-Weinberg equilibrium (HWE) *p* > 1 × 10^− 4^. Imputation was performed with the Michigan imputation server, combining phasing with eagle v2 and imputation with minimac 4, using 1000 Genome Project Phase 3 version 5 as haplotype reference panel [10–12]. Imputed data were filtered based on the genotype posterior probability (GP > 0.90). Following the imputation process, variants with a minor allele frequency (MAF) greater than 0.01 were retained for subsequent analyses [13].

### Low-coverage whole genome sequencing (lcWGS)

Sequencing libraries were generated using the Ultra II FS Library Prep Kit (New England Biolabs, Ipswich, USA). FFPE samples were pretreated using the NEBNext FFPE DNA Repair v2 Kit (New England Biolabs, Ipswich, Massachusetts, USA). Sequencing was performed at the NGS Core Facility at the University of Bonn (Bonn, Germany) using a S1 300 cycle flow cell on a Nova-Seq 6000 platform by Illumina Inc (San Diego, California, USA) in a paired-end 150 bp run. The raw sequencing reads were quality checked using MultiQC 1.14 [14]. Sequencing alignment was done using BWA-mem 0.7.17 and duplicates were removed using Picard 2.27.5 [15–17]. To achieve homogeneous genomic coverage across all samples, the sequencing data were downsampled in silico to a target coverage of 2x using Picard 2.27.5 [18]. Variant calling files were generated using GLIMPSE2 using 1000 Genome Project as haplotype reference [12, 19, 20]. Variants were subsequently filtered based on the genotype posterior probability (GP > 0.90) and reference population allele frequency (RAF > 0.01) using BCFtools 1.10.2 [21].

### Benchmarking

Prior to benchmarking all Variant Calling Format-Files (VCFs) were processed using pre.py (hap.py 1.0.0) allowing for normalized variant representation [22]. Benchmarking was done using vcfeval Rtg-tools (vcfeval) 3.12.1 [23, 24]. GIAB samples were benchmarked against publicly available GIAB reference files, whereas FFPE benchmarking was performed against the corresponding blood samples. Two parameters were used for benchmarking: (i) recall represents the proportion of all genotyped single nucleotide polymorphisms (SNPs) in FFPE tissue compared to blood samples and (ii) precision represents the proportion of correctly genotyped SNPs in FFPE tissue compared to blood samples [8].

$$\:Recall=\frac{True\:Positive}{True\:Positive+False\:Negative}$$

$$\:Precision=\frac{True\:Positive}{True\:Positive+False\:Positive}\:\:$$

### Statistical analysis

Statistical analyses were conducted using Graphpad Prism 6 (GraphPad Software Inc, Boston, Massachusetts, USA). Paired sample two-sided t-tests were performed to compare both genotyping approaches (microarray and lcWGS). A significance level of *p* < 0.05 was used to determine statistical significance.

## Results

### DNA quality assessment

Quality scores were computed for the GIAB, blood, and FFPE samples. The GIAB and blood samples exhibited similar metrics for the A260/280 ratio, the A260/230 ratio, the DNA integrity number (DIN), and the average fragment size (Table 1). Compared to the blood samples, FFPE samples showed a similar A260/280 ratio of 1.8 ± 0.0 (*p* = 1.000), but had a significantly lower A260/230 ratio of 0.9 ± 0.2 (*p* = 0.013). The DIN was also significantly lower at 5.5 ± 0.6 (*p* = 0.015). Furthermore, the average fragment size for the FFPE samples was 7,573.3 ± 1,811 bp, which was significantly smaller than that of the blood samples (*p* = 0.010) (Table 1).

**Table 1:** DNA quality scores stratified by sample type

Sample Type	A260/A280 [Mean ± SD]	A260/A230 [Mean ± SD]	DIN [Mean ± SD]	Average Size [bp] [Mean ± SD]	Call rate (Array) [Mean ± SD]
**GIAB**	1.9 ± 0	2.1 ± 0.1	8.7 ± 0.3	22,944 ± 468	0.996 ± 0.001
**Blood**	1.8 ± 0	2.1 ± 0.0	9.2 ± 0.1	22,574 ± 894	0.994 ± 0.001
**FFPE**	1.8 ± 0	0.9 ± 0.2	5.5 ± 0.6	7,573 ± 1,811	0.986 ± 0.005
p-value*	1.000	**0.013**	**0.015**	**0.010**	0.125

DIN: DNA Integrity Score, Average Size: Average fragment size in base pairs (bp). Call rate score applies only to microarray data. This table shows the mean and standard deviation (SD) of the different quality scores for each sample type. *p*-values were calculated using a two-sided paired-end t-test for the blood versus FFPE samples, with significance set at *p* < 0.05. Statistically significant differences are highlighted in bold

### Validation of microarrays and LcWGS

To assess the reliability of both genotyping pipelines, we performed benchmarking with three GIAB samples. This analysis revealed a recall of 0.772 ± 0.011 for microarray and 0.773 ± 0.006 for lcWGS, as well as a precision of 0.959 ± 0.010 for microarray and 0.994 ± 0.0004 for lcWGS (Table 2). Statistical comparison showed no significant difference in recall (*p* = 0.897), while precision was slightly higher for lcWGS (*p* = 0.035) (Table 2).

**Table 2:** Benchmarking summary

	Method	Recall [mean ± SD]	Precision [mean ± SD]	
**GIAB vs. Reference File** (Validation)	**Microarray**	0.772 ± 0.011	0.959 ± 0.010	
	**lcWGS**	0.773 ± 0.006	0.994 ± 0.0004	
	*p*-value	0.897	**0.035**	
**FFPE vs. Blood**	**Microarray**	0.999 ± 0.001	0.999 ± 0.001	
	**lcWGS**	0.969 ± 0.003	0.973 ± 0.001	
	*p*-value	**0.005**	**0.003**	

This table presents a comparative analysis of performance metrics between microarray and lcWGS. The upper section shows the results of the validation process (GIAB samples versus reference file), while the lower section displays the results of the FFPE tissue genotyping (FFPE samples versus blood samples). Recall and precision are reported as the mean values with their respective standard deviations (SD). Statistically significant differences between microarray and lcWGS (*p* < 0.05) are highlighted in bold

### Benchmarking: FFPE versus blood

Having assessed the reliability of both pipelines using GIAB samples, we further evaluated the suitability of FFPE samples for GWAS. For this purpose, FFPE samples were benchmarked against the corresponding blood samples for both genotyping methods.

**Fig. 1:**
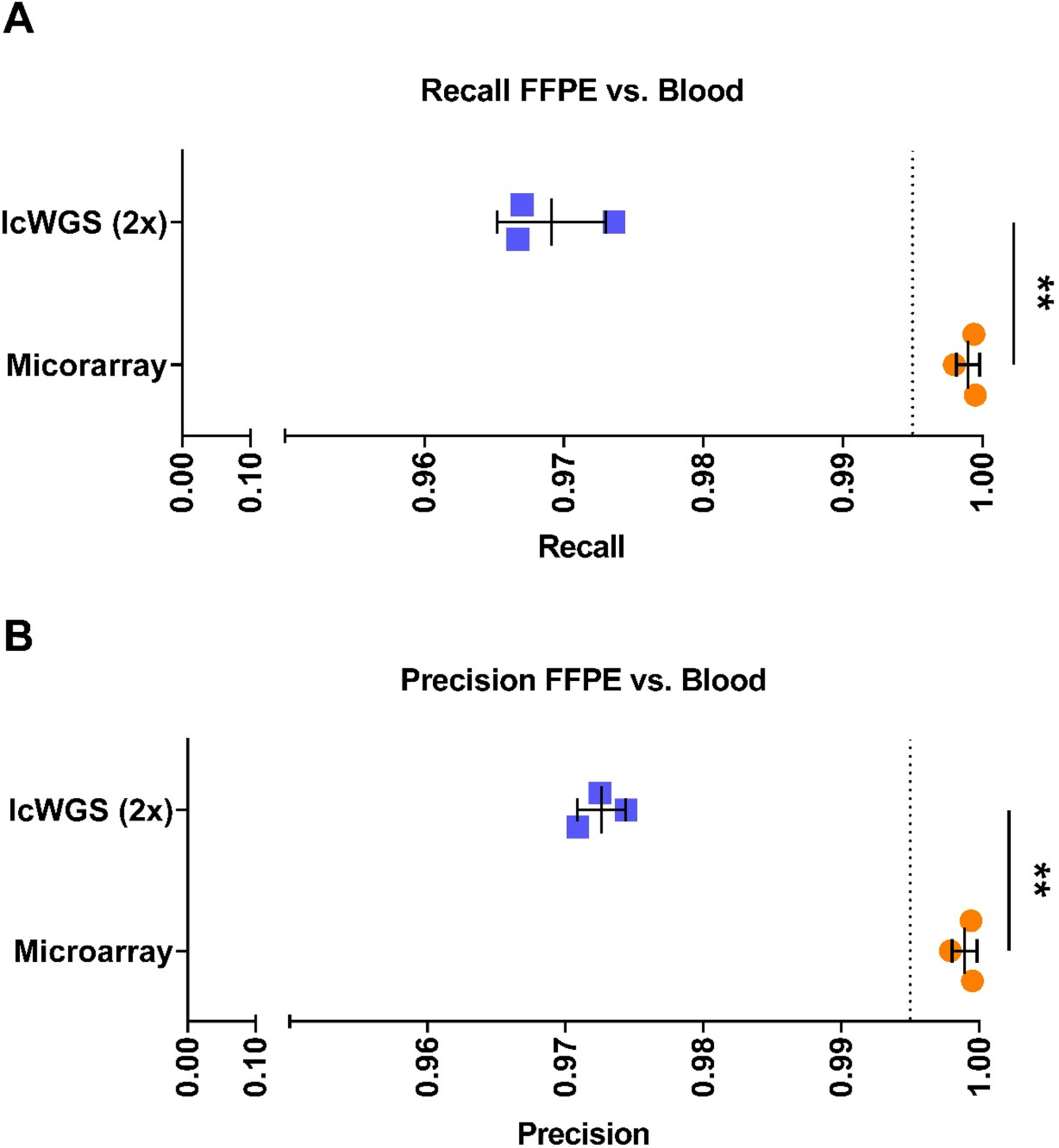
Comparison of microarray and lcWGS benchmarking. The recall (**A**) and precision values (**B**) of FFPE versus blood data sets were statistically analysed using a two-sided paired-end t-test. Legend: *p* > 0.05 (not significant (ns)), *p* < 0.05 (*), *p* < 0.01 (**), *p* < 0.001 (***), *p* < 0.0001 (****). The dotted line indicates a precision- /recall value of 99.5%

Microarray achieved a recall of 0.999 ± 0.001 and a precision of 0.999 ± 0.001, whereas lcWGS achieved a recall of 0.969 ± 0.003 and a precision of 0.973 ± 0.001 (Table 2; Fig. 1). Microarray demonstrated significantly higher recall (*p* = 0.005) and precision (*p* = 0.003) for FFPE genotyping compared to lcWGS (Table 2; Fig. 1). The comparison of SNP variant detection between the microarray and lcWGS approaches revealed significantly higher numbers of variants detected by our microarray workflow across all tissue types (Additional File 1). The microarray call rate for FFPE samples was 0.986 ± 0.005, while the call rates for blood and GIAB samples were 0.994 ± 0.001 and 0.996 ± 0.001, respectively. No significant difference was observed between FFPE and blood samples (*p* = 0.125) (Table 1).

## Discussion

The use of FFPE tissue in genetic research has gained increasing attention due to their wide availability in pathology institutes and the extensive clinical information associated with these samples [25].

### DNA quality analysis

Our analysis of DNA quality revealed expected results: while A260/280 ratios of FFPE samples were comparable to blood samples, the lower A260/230 ratios and DNA integrity numbers (DIN) indicated greater degradation and lower purity of FFPE-derived DNA. Furthermore, FFPE DNA exhibited significantly smaller average fragment sizes (Table 1). These metrics are expected for FFPE tissue as the formalin fixation process induces chemical modifications to DNA resulting in fragmentation and deamination [3]. These findings are consistent with previous studies confirming lower DNA quality in FFPE tissue but supporting its viability for genotyping [6, 26].

### Validation of genotyping methods

When validating our genotyping methods with GIAB samples, we observed high precision and moderate recall values (Table 2). This discrepancy in recall can be attributed to the comprehensive nature of the GIAB reference files, which contain a significant number of rare variants and INDELs that were not detected by our methods (Additional File 2) [8]. Both microarray and lcWGS were similarly affected by this. Notably, lcWGS demonstrated a significantly higher precision (Table 2), indicating its strength, when applied on high quality samples, as shown in large study cohorts [20]. Based on these observations, we conclude that both methods are reliable and suitable for further analysis using FFPE tissue. However, the application of our workflow may be limited in research contexts that require the detection of rare variants and INDELs. In particular for the lcWGS approach, the chosen coverage strongly affects the number of variants reported and should be adapted to the experimental setting.

### Reliability of FFPE samples

The genotyping concordance analysis between FFPE and blood samples showed that both microarray and lcWGS achieved high recall and precision values (Fig. 1; Table 2). This underlines the reliability of FFPE-derived DNA samples for GWAS, independently of the genotyping method. However, microarray outperformed lcWGS in terms of recall and precision (Fig. 1; Table 2). This discrepancy may be explained by the hypothesis that lcWGS is more prone to detecting residual formalin fixation artefacts, which could lead to an elevated false positive rate and a reduction in precision. Microarray technology, which is based on nucleic acid hybridization between immobilized probes and complementary target sequences, may be less susceptible to these artefacts, as evidenced by the high call rate observed in our study (Table 1) [27]. In contrast to genotyping arrays, which are constrained by ascertainment bias due to their dependence on pre-selected variants, lcWGS in theory is capable of detecting a more extensive range of genetic variation illustrating its potential for future research [28].

### Limitations

Despite these promising results, our analysis has several limitations. Differences in the formalin fixation method, type of sample collection (biopsy vs. resection), tissue origin, sample thickness, DNA-extraction method and storage conditions of FFPE samples could significantly affect the quality and integrity of the DNA extracted from FFPE tissues, thereby impacting the results of genotyping and their use for GWAS [29, 30]. In addition, as the samples used were formalin-fixed in 2021, our ability to assess the recall and precision of older FFPE samples, which may have experienced more DNA degradation over time, is limited [31]. This degradation, especially fragmentation and deamination, continues to be an obstacle. We have tried to overcome these by using specialised commercial kits for DNA extraction and NGS preparation. Another possibility would be the use of bioinformatic methods, such as the FFPEsig model developed by Guo et al. [32], which recognises the specific mutational signatures of FFPE samples and filters out false positives. This approach could further improve the results. This is especially true when a large number of FFPE samples are included in a GWAS.

Furthermore, our study did not have access to high-coverage sequencing data as a reference to compare lcWGS and array-based genotyping on FFPE samples. Because of this limitation, we used precision and recall—calculated relative to the blood genotyping data—as our primary evaluation metrics. Future studies with larger cohorts and high-coverage data could further refine precision assessments and provide a more definitive evaluation of the optimal genotyping method for FFPE samples.

## Conclusion

In conclusion, FFPE samples stored in pathology institutes could significantly expand the available GWAS datasets and provide insights into previously difficult to study phenotypes. Regarding the most appropriate genotyping method for use with FFPE-tissue, microarrays and lcWGS both provided reliable genotype information, with microarray technology currently offering superior performance in terms of recall, precision and cost-effectiveness. However, as sequencing costs continue to fall, the potentially higher genomic resolution of lcWGS may make it more attractive for use with FFPE samples in the future.

## Electronic supplementary material

Below is the link to the electronic supplementary material.

**Supplementary Material 1**: **Additional File 1**

**Supplementary Material 2**: **Additional File 2**

**Supplementary Material 3**: **Additional File 3**

## Data Availability

The sequencing of the GIAB samples generated during this study are publicly available at the European Genome-phenome Archive (EGA) under the study accession number (EGAS00001008103). The GIAB reference data are publicly available from the GIAB consortium hosted by the National Institute of Standards and Technology (Gaithersburg, USA). Other datasets generated and analysed in this study are not publicly available due to data protection reasons but can be obtained from the corresponding author upon reasonable request.

## Abbreviations

bp: Base pair
DIN: DNA integrity number
EA: Oesophageal adenocarcinoma
FFPE: Formalin-fixed paraffin-embedded
GIAB: Genome in a bottle
GP: Genotype posterior probability
lcWGS: Low-coverage whole genome sequencing
MAF: Minor allele frequency
QC: Quality control
SNPs: Single nucleotide polymorphisms
VCF: Variant calling format
INDEL: Insertion and deletion
GWAS: Genome-wide association studies
