# Supplementary figures and images for: Assessing the suitability of formalin-fixed paraffin-embedded (FFPE) tissue for genome-wide association studies (GWAS)
Source: BMC Res Notes. 2025 Jul 1;18:254. doi: 10.1186/s13104-025-07306-z (PMC12211376; doi:10.1186/s13104-025-07306-z)

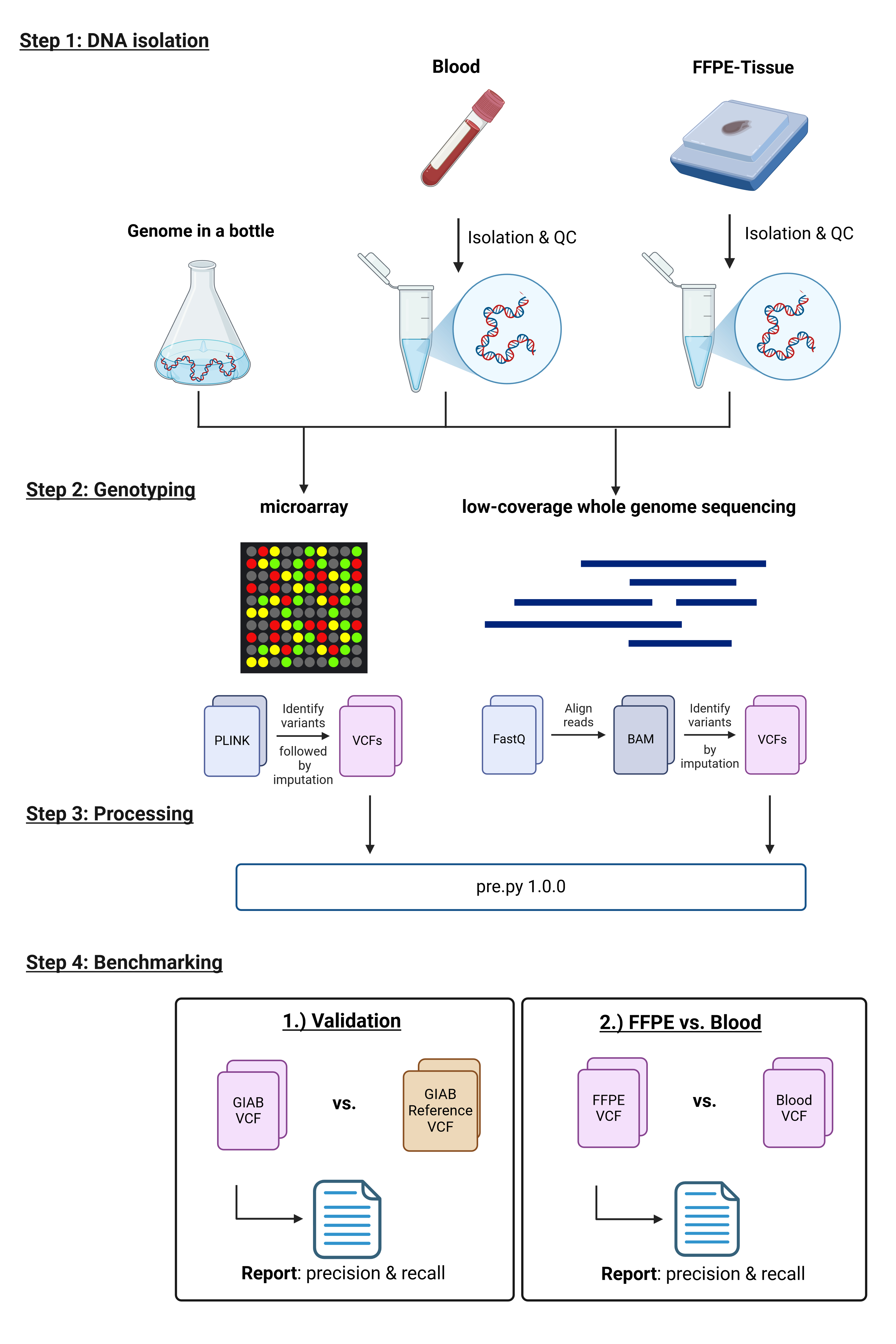

Supplement: Supplementary file 3 — Supplementary Material 3: Additional File 3 [file 13104_2025_7306_MOESM3_ESM.jpeg]
